# Synthesis and structure of an aryl­tellurenium(II) cation; [4-*tert*-butyl-2,6-bis­(1-pentyl-1*H*-benz­imidazol-2-yl-κ*N* ^3^)phenyl-κ*C* ^1^]tellurium(II) (1,4-dioxane)tri­iodido­mercurate(II)

**DOI:** 10.1107/S2056989018002645

**Published:** 2018-02-23

**Authors:** Varsha Rani, Harkesh B. Singh, Ray J. Butcher

**Affiliations:** a Department of Chemistry, Indian Institute of Technology Bombay, Powai, Mumbai 400076, India; b Department of Chemistry, Howard University, 525 College Street NW, Washington, DC 20059, USA

**Keywords:** crystal structure, aryl­tellurenium(II) cation, (1,4-dioxane)tri­iodo­mercury(II) anion

## Abstract

In the title salt, (C_34_H_41_N_4_Te)[HgI_3_(C_4_H_8_O_2_)], the aryl­tellurenium [C_34_H_41_N_4_Te]^+^ cations and [HgI_3_(dioxane)]^−^ anions are linked by a short inter­action between the Te atom and one of the I-atom donors of the anion, as well as through weak C—H⋯I inter­actions. The geometry around the Te atom is T-shaped with the coordination comprising a C atom of the central aromatic ring and two N atom donors of the benzimidazolyl moiety. The Te—N bond lengths are almost equal [2.232 (3) and 2.244 (3) Å], while the Te—C bond length is 2.071 (4) Å. The N—Te—N bond angle is 150.68 (11)°. The Hg^II^ atom of the anion is coordinated by iodide ions from three sides and the fourth coordination site is occupied by an O atom of the solvent mol­ecule (dioxane). Thus, it attains a trigonal–pyrimidal geometry, with O—Hg—I angles ranging of 90.76 (8) and 96.76 (7)° and I—Hg—I angles ranging from 112.41 (1) to 125.10 (1)°. The cations and anions are involved in numerous weak π–π stacking inter­actions involving both the central phenyl ring and two inversion-related benzimidazole moieties, which propagate in the *a*-axis direction. In addition, there are numerous C—H⋯I inter­actions between the cations and anions, which link them into a complex three-dimensional array.

## Abstract 2

In the structure of an aryl­tellurenium(II) salt containing a [4-*tert*-butyl-2,6-bis­(1-pentyl-1*H*-benzimidazol-2-yl)phen­yl]tellurium(II) cation and a [HgI_3_(dioxane)]^−^ anion, the cation and anion are linked by a C—H⋯I inter­action.

## Chemical context   

**Figure 1:**
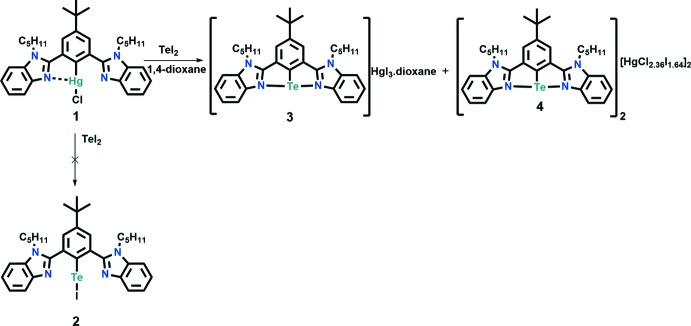
Reaction scheme.

Organoselenenium cations have been extensively studied and utilized in the area of synthetic organic chemistry (Back *et al.*, 1999 ▸; Singh & Wirth, 2012 ▸; Chivers & Laitinen, 2015 ▸), bio­logical fields (Mugesh & Singh, 2000 ▸; Singh & Wirth, 2012 ▸; Bhuyan & Mugesh, 2012 ▸) and material science (Manjare *et al.*, 2014 ▸; Kremer *et al.*, 2015 ▸). Compared to organoselenenium cations, the organotellurenium analogues are less studied. Fujihara *et al.* (1995 ▸) reported the first stable tellurenium cation, [{2,6-(Me_2_NCH_2_)_2_C_6_H_3_Te]^+^[PF_6_]^−^. After 16 years of preparation, the first structural characterization of the tellurenium cation [2,6-{O(CH_2_CH_2_)_2_NCH_2_}_2_C_6_H_3_Te]^+^[Hg_2_Cl_6_]^2−^ was demonstrated by Silvestru and co-workers (Beleaga *et al.*, 2011 ▸). Recently, we have reported the first examples of selone-stabilized aryl­tellurenium cations, which are synthesized by the reaction of mixed-valent tellurenyl bromide (*R*Te^II^–Te^IV^Br_2_–*R*) with 1,3-di­butyl­benzimidazolin-2-selone (Yadav *et al.*, 2016 ▸). While attempting to prepare a stable organotellurium iodide (**2**) (see Fig. 1 ▸) by the reaction of the mercury complex of 2,2′-(5-*tert*-butyl-1,3-phenyl­ene)bis­(1-pentyl-1*H*-benzimidazole) (C_34_H_41_N_4_HgCl) (**1**) with TeI_2_, the aryl­tellurenium cation [pent­yl(N^C^N)Te]^+^·[HgI_3_]^−^ (**3**) [N^C^N = 5-*tert*-butyl-1,3-bis­(*N*-pentyl benzimidazol-2′-yl)phen­yl)] was isolated as a by-product (3% yield) along with the major product, a dimeric aryl­tellurenium cation [C_34_H_41_N_4_Te]^+^_2_[HgCl_2.36_I_1.64_]^2−^ (**4**). The crystal structure of this compound is reported herein while the synthesis and structures of compounds **1** and **4** will be published elsewhere.
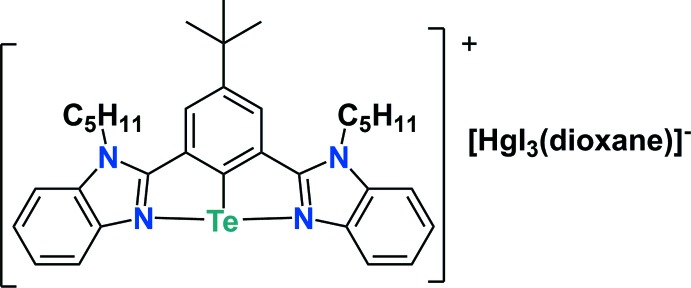


## Structural commentary   

**Figure 2:**
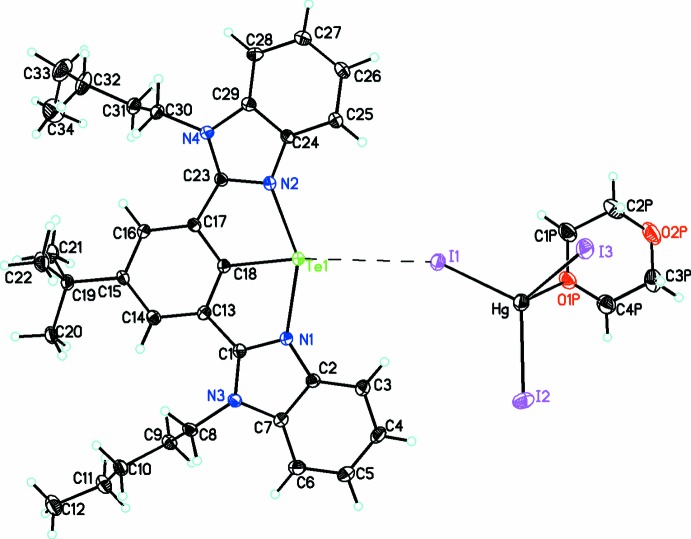
Diagram showing the atom-labeling scheme for the title compound, [C_34_H_41_N_4_Te]^+^[HgI_3_(dioxane)]^−^. The Te⋯I inter­action is shown as a dashed line. Displacement ellipsoids are drawn at the 30% probability level.

The title complex [C_34_H_41_N_4_Te]^+^[HgI_3_(dioxane)]^−^ is shown in Fig. 2 ▸. It crystallizes in *P*2_1_/*c* in the monoclinic crystal system. The asymmetric unit contains one tellurenium cationic unit stabilized by a [HgI_3_ (dioxane)]^−^ counter-anion. The coord­in­ation geometry around the Te atom is T-shaped whereby each Te atom is bonded with the central carbon atom of the aromatic ring and intra­molecularly coordinated with the two N atoms. This coordination gives rise to an octacyclic framework formed by two condensed five-membered rings, which is stable under ambient conditions. The observed Te—C bond length is 2.071 (4) Å, which is comparable with the related NCN pincer-based tellurenium cation in [2,6-{O(CH_2_CH_2_)_2_NCH_2_}_2_C_6_H_3_Te]^+^ [Hg_2_Cl_6_]^2−^ [2.074 (8) Å; Beleaga *et al.*, 2011 ▸]. The Te—N bond lengths are almost equal [2.232 (3) and 2.244 (3) Å]. The Te—N distances are shorter than the sum of the van der Waals radii for Te and N [Σ*r*vdw(Te,N) = 3.61 Å] and longer than the covalent radii [Σ*r*cov(Te,N) = 2.09 Å] (Bondi, 1964 ▸). This implies strong intra­molecular Te⋯N inter­actions in the tellurenium cation.

In the anion, the mercury atom is coordinated by three iodide ions and one oxygen atom from the solvent mol­ecule (1,4-dioxane), with Hg—I bond lengths of 2.6828 (4), 2.6912 (4) and 2.7321 (3) Å, which are in the range expected for an Hg—I covalent bond (the sum of the covalent radii of Hg and I is 2.71 Å). The Hg—O bond length of 2.730 (3) Å is longer than the sum of their covalent radii (2.15 Å), but shorter than the sum of their van der Waals radii (3.07 Å). This value is in the range found for previous Hg–dioxane structures [2.64 (1) to 2.83 (1) Å; Small, 1982 ▸; Frey & Monier, 1971 ▸; Crochet & Fromm, 2011 ▸]. The O—Hg—I bond angles are 90.76 (8) , 95.08 (7) and 96.76 (7)° and the I—Hg—I bond angles range from 112.41 (1) to 125.10 (1)°. The resulting geometry around the mercury atom is thus trigonal pyramidal with the Hg atom displaced by only 0.2018 (3) Å from the plane of the three I atoms, with the longer Hg—O bond at the apex of this pyramid.

In the 4-*tert*-butyl-2,6-bis­(1-pentyl-1*H*-benzimidazol-2-yl)phenyl ligand, the two pentyl substituents have adopted two different conformations. One has the normal extended zigzag conformation as shown by the N—C—C—C and C—C—C—C torsion angles [−173.8 (3), −173.5 (4) and −174.6 (4)°, respectively] while for the other, these angles are significantly different [−178.7 (4), 171.3 (5) and 66.0 (8), respectively]. In the central aromatic region, both benzimidazole moieties are almost coplanar with the central phenyl ring [dihedral angles of 5.3 (3) and 1.6 (2)°].

## Supra­molecular features   

**Figure 3:**
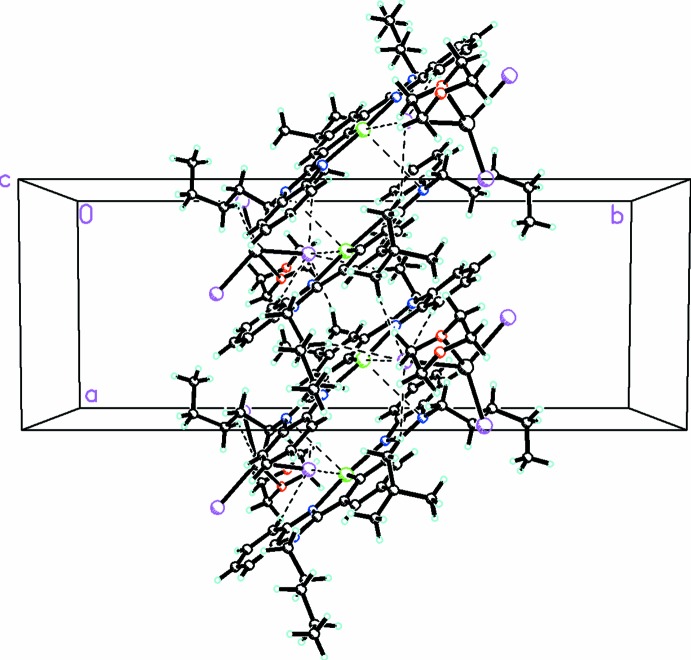
Packing diagram viewed along the *c*-axis direction, showing the parallel stacking of the cations. C—H⋯I and Te⋯I inter­actions are shown as dashed lines.

The mol­ecules are involved in numerous weak π–π stacking inter­actions involving both the central phenyl ring and the two benzimidazole moieties (symmetry code −*x* + 1, −*y* + 1, −*z* + 1), which propagate in the *a*-axis direction, as shown in Fig. 3 ▸. The shortest separation, however, is 3.4980 (19) Å between the centroid of one of the outer phenyl rings (C24–C29; symmetry code −*x* + 2, −*y* + 1, −*z* + 1) and the centroid of the moiety made up of the central phenyl ring and one of the imidazole rings (Te1/N1/N3/C1/C2/C7/C13–C18). There is a short inter­action between the Te atom and one of the iodine donors from the anion [Te1⋯I1 = 3.8859 (4) Å]. In addition there are numerous C—H⋯I inter­actions between the cations and anions (Table 1 ▸), which link them into a complex three-dimensional array (Fig. 3 ▸).

## Database survey   

A survey of the Cambridge Structural Database (web CSD version 1.19 with updates June 2017; Groom *et al.*, 2016 ▸) reveals that there is no structure report in the literature for a tellurenium cation with bis-benzimidazole moieties, although an NCN pincer-framework-based tellurenium cation has one hit (Beleaga *et al.*, 2011 ▸). There are four reports in the literature of structures involving Hg coordinated to dioxane (BIYPAA, Small, 1982 ▸; HGBDOX, Frey & Monier, 1971 ▸; VALRUX and VALSAE, Crochet & Fromm, 2011 ▸), including one which also contains Hg—I bonds (VALSAE, Crochet & Fromm, 2011 ▸).

## Synthesis and crystallization   

The reaction scheme for the synthesis of the title compound is shown in Fig. 1 ▸. To a solution of **1** (0.2 g, 0.269 mmol) in 1,4-dioxane (60 ml) was added 0.102 g of TeI_2_ were added. The reaction mixture was stirred for 24 h at room temperature in an inert atmosphere. The reaction mixture was filtered. The filtrate was evaporated and reduced to 5 mL. Colorless prismatic crystals were obtained from slow evaporation of a1,4-dioxane solution of the compound at room temperature.

Yield 3% (0.035 g). HR–MS: *m*/*z* calculated for C_34_H_41_N_4_Te is 635.2394. Found 635.2391. ESI–MS (negative mode): *m*/*z* calculated for HgI_3_: 582.6840. Found 582.6543

## Refinement   

Crystal data, data collection and structure refinement details are summarized in Table 2 ▸. The H atoms were positioned geometrically, with C—H = ranging from 0.95 to 0.99 Å, and allowed to ride on their parent atoms with *U*_iso_(H) = *xU*_eq_(C), where *x* = 1.5 for methyl H atoms and 1.2 for all other C-bound H atoms.

## Supplementary Material

Crystal structure: contains datablock(s) I. DOI: 10.1107/S2056989018002645/zs2398sup1.cif

Structure factors: contains datablock(s) I. DOI: 10.1107/S2056989018002645/zs2398Isup2.hkl

CCDC reference: 1823822

Additional supporting information:  crystallographic information; 3D view; checkCIF report

## Figures and Tables

**Table 1:** Hydrogen-bond geometry (Å, °)

*D*—H⋯*A*	*D*—H	H⋯*A*	*D*⋯*A*	*D*—H⋯*A*
C3—H3*A*⋯I1	0.95	3.16	4.086 (4)	164
C8—H8*B*⋯I2^i^	0.99	3.08	3.997 (4)	155
C9—H9*B*⋯I1^ii^	0.99	3.18	4.095 (4)	155
C30—H30*B*⋯I1^iii^	0.99	3.28	4.111 (4)	142
C31—H31*B*⋯I3^iv^	0.99	3.20	4.185 (4)	172

Symmetry codes: (i) 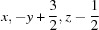; (ii) 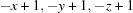; (iii) 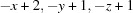; (iv) 
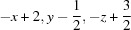
.

**Table 2:** Experimental details

Crystal data	
Chemical formula	(C_34_H_41_N_4_Te)[HgI_3_(C_4_H_8_O_2_)]
*M*_r_	1302.70
Crystal system, space group	Monoclinic, *P*2_1_/*c*
Temperature (K)	100
*a*, *b*, *c* (Å)	9.6074 (2), 25.4943 (6), 17.4326 (3)
β (°)	95.152 (2)
*V* (Å^3^)	4252.59 (15)
*Z*	4
Radiation type	Mo *K*α
μ (mm^−1^)	6.51
Crystal size (mm)	0.27 × 0.21 × 0.13

Data collection	
Diffractometer	Rigaku CCD dual source
Absorption correction	Multi-scan (*CrysAlis PRO*; Rigaku OD, 2015 ▸)
*T*_min_, *T*_max_	0.238, 0.567
No. of measured, independent and observed [*I* > 2σ(*I*)] reflections	58708, 12625, 11288
*R*_int_	0.041
(sin θ/λ)_max_ (Å^−1^)	0.728

Refinement	
*R*[*F* ^2^ > 2σ(*F* ^2^)], *wR*(*F* ^2^), *S*	0.041, 0.080, 1.14
No. of reflections	12625
No. of parameters	448
H-atom treatment	H-atom parameters constrained
Δρ_max_, Δρ_min_ (e Å^−3^)	1.45, −1.53

Computer programs: *CrysAlis PRO* (Rigaku OD, 2015 ▸), *SHELXT* (Sheldrick, 2015*a* ▸), *SHELXL2017* (Sheldrick, 2015*b* ▸) and *SHELXTL* (Sheldrick, 2008 ▸).

## supplementary crystallographic information

### Crystal data

**Table:**

(C_34_H_41_N_4_Te)[HgI_3_(C_4_H_8_O_2_)]	*F*(000) = 2448
*M**_r_* = 1302.70	*D*_x_ = 2.035 Mg m^−^^3^
Monoclinic, *P*2_1_/*c*	Mo *K*α radiation, λ = 0.71073 Å
*a* = 9.6074 (2) Å	Cell parameters from 22849 reflections
*b* = 25.4943 (6) Å	θ = 2.0–31.3°
*c* = 17.4326 (3) Å	µ = 6.51 mm^−^^1^
β = 95.152 (2)°	*T* = 100 K
*V* = 4252.59 (15) Å^3^	Prism, colorless
*Z* = 4	0.27 × 0.21 × 0.13 mm

### Data collection

**Table:**

Rigaku CCD dual source diffractometer	11288 reflections with *I* > 2σ(*I*)
Radiation source: fine-focus sealed X-ray tube	*R*_int_ = 0.041
ω scans	θ_max_ = 31.2°, θ_min_ = 2.1°
Absorption correction: multi-scan (CrysAlis PRO; Rigaku OD, 2015)	*h* = −13→13
*T*_min_ = 0.238, *T*_max_ = 0.567	*k* = −34→36
58708 measured reflections	*l* = −25→25
12625 independent reflections	

### Refinement

**Table:**

Refinement on *F*^2^	0 restraints
Least-squares matrix: full	Hydrogen site location: inferred from neighbouring sites
*R*[*F*^2^ > 2σ(*F*^2^)] = 0.041	H-atom parameters constrained
*wR*(*F*^2^) = 0.080	*w* = 1/[σ^2^(*F*_o_^2^) + (0.0267*P*)^2^ + 9.9235*P*] where *P* = (*F*_o_^2^ + 2*F*_c_^2^)/3
*S* = 1.14	(Δ/σ)_max_ = 0.004
12625 reflections	Δρ_max_ = 1.45 e Å^−^^3^
448 parameters	Δρ_min_ = −1.53 e Å^−^^3^

### Special details

**Table:**

Geometry. All esds (except the esd in the dihedral angle between two l.s. planes) are estimated using the full covariance matrix. The cell esds are taken into account individually in the estimation of esds in distances, angles and torsion angles; correlations between esds in cell parameters are only used when they are defined by crystal symmetry. An approximate (isotropic) treatment of cell esds is used for estimating esds involving l.s. planes.
Refinement. Refined as a 2-component twin.

### Fractional atomic coordinates and isotropic or equivalent isotropic displacement parameters (Å^2^)

**Table:**

	*x*	*y*	*z*	*U*_iso_*/*U*_eq_	
Hg	0.75646 (2)	0.67318 (2)	0.82692 (2)	0.03297 (5)	
I1	0.73560 (3)	0.57972 (2)	0.75006 (2)	0.03369 (7)	
I2	0.55214 (3)	0.74060 (2)	0.77764 (2)	0.04473 (8)	
I3	0.99100 (3)	0.69905 (2)	0.91390 (2)	0.04466 (8)	
O1P	0.6255 (4)	0.63149 (14)	0.94549 (18)	0.0431 (8)	
O2P	0.6836 (5)	0.6252 (2)	1.1071 (2)	0.0653 (12)	
C1P	0.6860 (6)	0.5855 (2)	0.9821 (3)	0.0491 (13)	
H1PA	0.747374	0.568080	0.947248	0.059*	
H1PB	0.610890	0.560639	0.992658	0.059*	
C2P	0.7689 (6)	0.5994 (3)	1.0559 (3)	0.0519 (13)	
H2PA	0.808972	0.567185	1.080570	0.062*	
H2PB	0.847183	0.622757	1.044987	0.062*	
C3P	0.6282 (8)	0.6717 (3)	1.0710 (4)	0.0686 (19)	
H3PA	0.705702	0.695483	1.060347	0.082*	
H3PB	0.568885	0.690112	1.106058	0.082*	
C4P	0.5428 (6)	0.6587 (3)	0.9969 (3)	0.0525 (14)	
H4PA	0.462352	0.636580	1.007958	0.063*	
H4PB	0.506000	0.691438	0.972173	0.063*	
Te1	0.74063 (2)	0.51396 (2)	0.54923 (2)	0.02140 (5)	
N1	0.5897 (3)	0.56893 (13)	0.48474 (18)	0.0236 (6)	
N2	0.8934 (3)	0.44789 (13)	0.55087 (18)	0.0237 (6)	
N3	0.4931 (3)	0.59799 (13)	0.37156 (18)	0.0244 (6)	
N4	1.0026 (3)	0.38656 (13)	0.48812 (18)	0.0241 (6)	
C1	0.5772 (4)	0.56054 (15)	0.4084 (2)	0.0228 (7)	
C2	0.5088 (4)	0.61244 (15)	0.4987 (2)	0.0247 (7)	
C3	0.4854 (4)	0.63775 (18)	0.5677 (3)	0.0324 (9)	
H3A	0.526433	0.625558	0.616065	0.039*	
C4	0.3999 (5)	0.68122 (18)	0.5620 (3)	0.0364 (10)	
H4A	0.383711	0.699948	0.607423	0.044*	
C5	0.3358 (5)	0.69872 (19)	0.4909 (3)	0.0387 (10)	
H5A	0.275877	0.728408	0.489732	0.046*	
C6	0.3578 (5)	0.67387 (17)	0.4230 (3)	0.0334 (9)	
H6A	0.314010	0.685579	0.374971	0.040*	
C7	0.4471 (4)	0.63077 (15)	0.4278 (2)	0.0259 (8)	
C8	0.4633 (4)	0.60656 (16)	0.2885 (2)	0.0267 (8)	
H8A	0.543550	0.593917	0.261713	0.032*	
H8B	0.453223	0.644667	0.278569	0.032*	
C9	0.3321 (4)	0.57888 (17)	0.2554 (2)	0.0288 (8)	
H9A	0.250131	0.594283	0.277669	0.035*	
H9B	0.337518	0.541345	0.269913	0.035*	
C10	0.3127 (4)	0.58365 (19)	0.1674 (2)	0.0322 (9)	
H10A	0.318795	0.621078	0.152928	0.039*	
H10B	0.389474	0.564727	0.145069	0.039*	
C11	0.1732 (5)	0.5616 (2)	0.1334 (3)	0.0397 (11)	
H11A	0.096416	0.582704	0.151795	0.048*	
H11B	0.163106	0.525180	0.151965	0.048*	
C12	0.1598 (6)	0.5616 (2)	0.0453 (3)	0.0506 (13)	
H12A	0.070863	0.545423	0.026178	0.076*	
H12B	0.237218	0.541619	0.026742	0.076*	
H12C	0.162796	0.597783	0.026544	0.076*	
C13	0.6514 (4)	0.51672 (15)	0.3791 (2)	0.0225 (7)	
C14	0.6451 (4)	0.49851 (16)	0.3031 (2)	0.0250 (7)	
H14A	0.583208	0.515082	0.264960	0.030*	
C15	0.7270 (4)	0.45675 (16)	0.2818 (2)	0.0237 (7)	
C16	0.8152 (4)	0.43160 (15)	0.3391 (2)	0.0246 (7)	
H16A	0.871573	0.403114	0.325237	0.029*	
C17	0.8217 (4)	0.44771 (15)	0.4163 (2)	0.0221 (7)	
C18	0.7395 (4)	0.49037 (14)	0.4355 (2)	0.0215 (7)	
C19	0.7261 (4)	0.43807 (17)	0.1985 (2)	0.0275 (8)	
C20	0.6176 (5)	0.46742 (19)	0.1444 (2)	0.0337 (9)	
H20A	0.636546	0.505170	0.147362	0.050*	
H20B	0.523754	0.460497	0.159841	0.050*	
H20C	0.623314	0.455319	0.091394	0.050*	
C21	0.6910 (6)	0.37930 (19)	0.1935 (3)	0.0427 (11)	
H21A	0.604471	0.372690	0.217678	0.064*	
H21B	0.767486	0.359149	0.220351	0.064*	
H21C	0.678832	0.368573	0.139351	0.064*	
C22	0.8719 (5)	0.4479 (2)	0.1713 (3)	0.0402 (11)	
H22A	0.894545	0.485269	0.175965	0.060*	
H22B	0.872508	0.437028	0.117394	0.060*	
H22C	0.941604	0.427499	0.203245	0.060*	
C23	0.9077 (4)	0.42570 (14)	0.4821 (2)	0.0223 (7)	
C24	0.9865 (4)	0.42250 (15)	0.6039 (2)	0.0232 (7)	
C25	1.0173 (4)	0.43169 (16)	0.6829 (2)	0.0263 (8)	
H25A	0.972775	0.458794	0.709028	0.032*	
C26	1.1166 (4)	0.39891 (17)	0.7210 (2)	0.0302 (8)	
H26A	1.141048	0.403739	0.774576	0.036*	
C27	1.1814 (4)	0.35895 (17)	0.6821 (2)	0.0304 (8)	
H27A	1.247151	0.336915	0.710397	0.036*	
C28	1.1528 (4)	0.35036 (16)	0.6033 (2)	0.0281 (8)	
H28A	1.198365	0.323620	0.577025	0.034*	
C29	1.0539 (4)	0.38315 (15)	0.5655 (2)	0.0236 (7)	
C30	1.0625 (4)	0.35688 (16)	0.4263 (2)	0.0277 (8)	
H30A	1.160849	0.348098	0.443315	0.033*	
H30B	1.062647	0.379541	0.380238	0.033*	
C31	0.9845 (5)	0.30670 (16)	0.4042 (2)	0.0308 (9)	
H31A	0.886719	0.315002	0.385220	0.037*	
H31B	0.982848	0.283726	0.449883	0.037*	
C32	1.0563 (6)	0.2783 (2)	0.3413 (3)	0.0481 (13)	
H32A	1.157460	0.275633	0.357500	0.058*	
H32B	1.045373	0.299530	0.293597	0.058*	
C33	0.9982 (7)	0.2230 (3)	0.3236 (4)	0.0668 (18)	
H33A	1.057355	0.205345	0.287666	0.080*	
H33B	1.003942	0.202324	0.371852	0.080*	
C34	0.8516 (8)	0.2232 (3)	0.2892 (4)	0.0698 (18)	
H34A	0.826398	0.188126	0.269610	0.105*	
H34B	0.841711	0.248498	0.246670	0.105*	
H34C	0.789684	0.233200	0.328424	0.105*	

### Atomic displacement parameters (Å^2^)

**Table:**

	*U*^11^	*U*^22^	*U*^33^	*U*^12^	*U*^13^	*U*^23^
Hg	0.03525 (9)	0.03204 (9)	0.03157 (8)	−0.00056 (7)	0.00279 (6)	−0.00291 (6)
I1	0.03544 (14)	0.03364 (14)	0.03205 (14)	0.00098 (11)	0.00334 (11)	−0.00527 (11)
I2	0.04247 (17)	0.02744 (14)	0.0635 (2)	0.00214 (12)	0.00065 (15)	0.00296 (14)
I3	0.04082 (16)	0.04536 (18)	0.04619 (18)	−0.00267 (14)	−0.00499 (13)	−0.01225 (14)
O1P	0.0482 (19)	0.053 (2)	0.0277 (16)	0.0038 (16)	0.0029 (14)	0.0056 (14)
O2P	0.071 (3)	0.094 (3)	0.0289 (18)	0.004 (2)	−0.0056 (18)	0.002 (2)
C1P	0.061 (3)	0.049 (3)	0.036 (3)	−0.003 (3)	−0.003 (2)	0.009 (2)
C2P	0.048 (3)	0.068 (4)	0.038 (3)	−0.003 (3)	−0.003 (2)	0.013 (3)
C3P	0.079 (5)	0.083 (5)	0.042 (3)	0.013 (4)	0.001 (3)	−0.018 (3)
C4P	0.048 (3)	0.072 (4)	0.039 (3)	0.012 (3)	0.007 (2)	0.000 (3)
Te1	0.02258 (11)	0.02243 (11)	0.01920 (10)	−0.00065 (9)	0.00201 (8)	−0.00145 (9)
N1	0.0245 (15)	0.0244 (16)	0.0216 (15)	0.0020 (13)	−0.0003 (12)	−0.0026 (12)
N2	0.0256 (15)	0.0259 (16)	0.0197 (14)	0.0010 (13)	0.0020 (12)	−0.0012 (12)
N3	0.0244 (15)	0.0264 (16)	0.0221 (15)	0.0016 (13)	0.0010 (12)	0.0024 (12)
N4	0.0247 (15)	0.0232 (15)	0.0243 (15)	0.0022 (12)	0.0012 (12)	−0.0006 (12)
C1	0.0221 (17)	0.0243 (18)	0.0223 (17)	−0.0017 (14)	0.0034 (13)	−0.0005 (14)
C2	0.0232 (17)	0.0243 (18)	0.0264 (18)	0.0011 (14)	0.0017 (14)	−0.0004 (14)
C3	0.032 (2)	0.036 (2)	0.030 (2)	0.0043 (18)	0.0048 (16)	−0.0031 (17)
C4	0.037 (2)	0.035 (2)	0.039 (2)	0.0091 (19)	0.0088 (19)	−0.0087 (19)
C5	0.039 (2)	0.036 (2)	0.041 (2)	0.013 (2)	0.006 (2)	−0.002 (2)
C6	0.032 (2)	0.033 (2)	0.036 (2)	0.0047 (18)	0.0031 (17)	0.0045 (18)
C7	0.0255 (18)	0.0245 (18)	0.0281 (19)	0.0016 (15)	0.0039 (15)	−0.0010 (15)
C8	0.0297 (19)	0.0264 (19)	0.0241 (18)	0.0024 (16)	0.0034 (15)	0.0070 (15)
C9	0.0267 (19)	0.035 (2)	0.0250 (18)	0.0000 (16)	0.0023 (15)	0.0045 (16)
C10	0.032 (2)	0.042 (2)	0.0234 (18)	0.0032 (18)	0.0037 (16)	0.0030 (17)
C11	0.035 (2)	0.056 (3)	0.027 (2)	−0.003 (2)	−0.0016 (17)	0.002 (2)
C12	0.053 (3)	0.068 (4)	0.029 (2)	0.000 (3)	−0.003 (2)	0.001 (2)
C13	0.0205 (16)	0.0227 (17)	0.0248 (17)	−0.0014 (14)	0.0050 (13)	−0.0006 (14)
C14	0.0226 (17)	0.0292 (19)	0.0230 (17)	−0.0020 (15)	0.0011 (14)	0.0015 (14)
C15	0.0241 (17)	0.0299 (19)	0.0169 (16)	−0.0014 (15)	0.0008 (13)	0.0011 (14)
C16	0.0271 (18)	0.0232 (18)	0.0234 (17)	0.0022 (15)	0.0026 (14)	−0.0033 (14)
C17	0.0229 (17)	0.0223 (17)	0.0209 (17)	−0.0019 (14)	0.0010 (13)	0.0011 (13)
C18	0.0236 (17)	0.0207 (17)	0.0204 (16)	−0.0022 (14)	0.0028 (13)	0.0001 (13)
C19	0.0274 (19)	0.033 (2)	0.0216 (17)	0.0010 (16)	0.0011 (14)	−0.0054 (15)
C20	0.032 (2)	0.048 (3)	0.0198 (18)	0.0035 (19)	0.0007 (16)	−0.0041 (17)
C21	0.054 (3)	0.040 (3)	0.033 (2)	0.002 (2)	−0.002 (2)	−0.015 (2)
C22	0.029 (2)	0.065 (3)	0.027 (2)	0.001 (2)	0.0046 (17)	−0.005 (2)
C23	0.0254 (17)	0.0214 (17)	0.0200 (16)	−0.0004 (14)	0.0016 (13)	0.0020 (13)
C24	0.0229 (17)	0.0234 (18)	0.0229 (17)	−0.0023 (14)	0.0000 (13)	0.0031 (14)
C25	0.0239 (18)	0.031 (2)	0.0238 (18)	−0.0040 (15)	0.0023 (14)	0.0003 (15)
C26	0.032 (2)	0.036 (2)	0.0222 (18)	−0.0047 (17)	−0.0014 (15)	0.0053 (16)
C27	0.0265 (19)	0.033 (2)	0.032 (2)	0.0013 (16)	0.0002 (16)	0.0073 (17)
C28	0.0282 (19)	0.0241 (19)	0.032 (2)	0.0035 (16)	0.0010 (16)	0.0039 (16)
C29	0.0225 (17)	0.0242 (18)	0.0241 (17)	−0.0018 (14)	0.0029 (14)	0.0044 (14)
C30	0.0308 (19)	0.0263 (19)	0.0266 (18)	0.0050 (16)	0.0062 (15)	−0.0016 (15)
C31	0.038 (2)	0.0251 (19)	0.030 (2)	−0.0003 (17)	0.0081 (17)	−0.0023 (16)
C32	0.051 (3)	0.043 (3)	0.053 (3)	−0.007 (2)	0.019 (2)	−0.024 (2)
C33	0.069 (4)	0.056 (4)	0.076 (4)	0.008 (3)	0.005 (3)	−0.036 (3)
C34	0.080 (5)	0.056 (4)	0.070 (4)	−0.014 (3)	−0.006 (4)	−0.013 (3)

### Geometric parameters (Å, º)

**Table:**

Hg—I3	2.6828 (4)	C11—H11A	0.9900
Hg—I2	2.6912 (4)	C11—H11B	0.9900
Hg—O1P	2.730 (3)	C12—H12A	0.9800
Hg—I1	2.7321 (3)	C12—H12B	0.9800
O1P—C4P	1.429 (6)	C12—H12C	0.9800
O1P—C1P	1.433 (6)	C13—C14	1.400 (5)
O2P—C3P	1.424 (8)	C13—C18	1.409 (5)
O2P—C2P	1.425 (7)	C14—C15	1.394 (6)
C1P—C2P	1.494 (7)	C14—H14A	0.9500
C1P—H1PA	0.9900	C15—C16	1.405 (5)
C1P—H1PB	0.9900	C15—C19	1.527 (5)
C2P—H2PA	0.9900	C16—C17	1.404 (5)
C2P—H2PB	0.9900	C16—H16A	0.9500
C3P—C4P	1.504 (8)	C17—C18	1.402 (5)
C3P—H3PA	0.9900	C17—C23	1.464 (5)
C3P—H3PB	0.9900	C19—C20	1.536 (6)
C4P—H4PA	0.9900	C19—C21	1.536 (6)
C4P—H4PB	0.9900	C19—C22	1.540 (6)
Te1—C18	2.071 (4)	C20—H20A	0.9800
Te1—N2	2.232 (3)	C20—H20B	0.9800
Te1—N1	2.244 (3)	C20—H20C	0.9800
N1—C1	1.343 (5)	C21—H21A	0.9800
N1—C2	1.389 (5)	C21—H21B	0.9800
N2—C23	1.344 (5)	C21—H21C	0.9800
N2—C24	1.387 (5)	C22—H22A	0.9800
N3—C1	1.372 (5)	C22—H22B	0.9800
N3—C7	1.391 (5)	C22—H22C	0.9800
N3—C8	1.467 (5)	C24—C29	1.396 (5)
N4—C23	1.349 (5)	C24—C25	1.403 (5)
N4—C29	1.397 (5)	C25—C26	1.391 (6)
N4—C30	1.475 (5)	C25—H25A	0.9500
C1—C13	1.443 (5)	C26—C27	1.400 (6)
C2—C3	1.401 (6)	C26—H26A	0.9500
C2—C7	1.402 (5)	C27—C28	1.394 (6)
C3—C4	1.378 (6)	C27—H27A	0.9500
C3—H3A	0.9500	C28—C29	1.386 (5)
C4—C5	1.406 (7)	C28—H28A	0.9500
C4—H4A	0.9500	C30—C31	1.515 (6)
C5—C6	1.376 (6)	C30—H30A	0.9900
C5—H5A	0.9500	C30—H30B	0.9900
C6—C7	1.392 (6)	C31—C32	1.529 (6)
C6—H6A	0.9500	C31—H31A	0.9900
C8—C9	1.513 (6)	C31—H31B	0.9900
C8—H8A	0.9900	C32—C33	1.538 (8)
C8—H8B	0.9900	C32—H32A	0.9900
C9—C10	1.534 (5)	C32—H32B	0.9900
C9—H9A	0.9900	C33—C34	1.481 (9)
C9—H9B	0.9900	C33—H33A	0.9900
C10—C11	1.524 (6)	C33—H33B	0.9900
C10—H10A	0.9900	C34—H34A	0.9800
C10—H10B	0.9900	C34—H34B	0.9800
C11—C12	1.529 (6)	C34—H34C	0.9800
			
I3—Hg—I2	125.097 (12)	C11—C12—H12C	109.5
I3—Hg—O1P	95.08 (7)	H12A—C12—H12C	109.5
I2—Hg—O1P	96.76 (7)	H12B—C12—H12C	109.5
I3—Hg—I1	120.825 (12)	C14—C13—C18	118.3 (3)
I2—Hg—I1	112.406 (11)	C14—C13—C1	127.6 (4)
O1P—Hg—I1	90.76 (8)	C18—C13—C1	114.0 (3)
C4P—O1P—C1P	110.1 (4)	C15—C14—C13	121.8 (4)
C4P—O1P—Hg	127.3 (3)	C15—C14—H14A	119.1
C1P—O1P—Hg	117.4 (3)	C13—C14—H14A	119.1
C3P—O2P—C2P	108.7 (4)	C14—C15—C16	118.6 (3)
O1P—C1P—C2P	110.7 (5)	C14—C15—C19	122.4 (3)
O1P—C1P—H1PA	109.5	C16—C15—C19	119.0 (3)
C2P—C1P—H1PA	109.5	C17—C16—C15	121.3 (4)
O1P—C1P—H1PB	109.5	C17—C16—H16A	119.3
C2P—C1P—H1PB	109.5	C15—C16—H16A	119.3
H1PA—C1P—H1PB	108.1	C18—C17—C16	118.6 (3)
O2P—C2P—C1P	110.9 (5)	C18—C17—C23	113.6 (3)
O2P—C2P—H2PA	109.5	C16—C17—C23	127.8 (3)
C1P—C2P—H2PA	109.5	C17—C18—C13	121.3 (3)
O2P—C2P—H2PB	109.5	C17—C18—Te1	119.9 (3)
C1P—C2P—H2PB	109.5	C13—C18—Te1	118.7 (3)
H2PA—C2P—H2PB	108.1	C15—C19—C20	112.2 (3)
O2P—C3P—C4P	110.4 (5)	C15—C19—C21	109.9 (3)
O2P—C3P—H3PA	109.6	C20—C19—C21	107.9 (4)
C4P—C3P—H3PA	109.6	C15—C19—C22	108.3 (3)
O2P—C3P—H3PB	109.6	C20—C19—C22	108.5 (4)
C4P—C3P—H3PB	109.6	C21—C19—C22	110.0 (4)
H3PA—C3P—H3PB	108.1	C19—C20—H20A	109.5
O1P—C4P—C3P	110.8 (5)	C19—C20—H20B	109.5
O1P—C4P—H4PA	109.5	H20A—C20—H20B	109.5
C3P—C4P—H4PA	109.5	C19—C20—H20C	109.5
O1P—C4P—H4PB	109.5	H20A—C20—H20C	109.5
C3P—C4P—H4PB	109.5	H20B—C20—H20C	109.5
H4PA—C4P—H4PB	108.1	C19—C21—H21A	109.5
C18—Te1—N2	74.93 (13)	C19—C21—H21B	109.5
C18—Te1—N1	75.77 (13)	H21A—C21—H21B	109.5
N2—Te1—N1	150.68 (11)	C19—C21—H21C	109.5
C1—N1—C2	107.4 (3)	H21A—C21—H21C	109.5
C1—N1—Te1	113.2 (2)	H21B—C21—H21C	109.5
C2—N1—Te1	139.2 (3)	C19—C22—H22A	109.5
C23—N2—C24	106.6 (3)	C19—C22—H22B	109.5
C23—N2—Te1	115.2 (2)	H22A—C22—H22B	109.5
C24—N2—Te1	138.1 (3)	C19—C22—H22C	109.5
C1—N3—C7	107.4 (3)	H22A—C22—H22C	109.5
C1—N3—C8	128.3 (3)	H22B—C22—H22C	109.5
C7—N3—C8	124.1 (3)	N2—C23—N4	111.4 (3)
C23—N4—C29	107.4 (3)	N2—C23—C17	116.2 (3)
C23—N4—C30	128.9 (3)	N4—C23—C17	132.4 (3)
C29—N4—C30	123.1 (3)	N2—C24—C29	108.4 (3)
N1—C1—N3	110.5 (3)	N2—C24—C25	130.0 (4)
N1—C1—C13	118.1 (3)	C29—C24—C25	121.6 (4)
N3—C1—C13	131.5 (3)	C26—C25—C24	116.3 (4)
N1—C2—C3	130.9 (4)	C26—C25—H25A	121.8
N1—C2—C7	108.1 (3)	C24—C25—H25A	121.8
C3—C2—C7	121.0 (4)	C25—C26—C27	121.4 (4)
C4—C3—C2	116.6 (4)	C25—C26—H26A	119.3
C4—C3—H3A	121.7	C27—C26—H26A	119.3
C2—C3—H3A	121.7	C28—C27—C26	122.4 (4)
C3—C4—C5	122.1 (4)	C28—C27—H27A	118.8
C3—C4—H4A	119.0	C26—C27—H27A	118.8
C5—C4—H4A	119.0	C29—C28—C27	116.1 (4)
C6—C5—C4	121.6 (4)	C29—C28—H28A	122.0
C6—C5—H5A	119.2	C27—C28—H28A	122.0
C4—C5—H5A	119.2	C28—C29—C24	122.2 (4)
C5—C6—C7	116.9 (4)	C28—C29—N4	131.6 (4)
C5—C6—H6A	121.6	C24—C29—N4	106.2 (3)
C7—C6—H6A	121.6	N4—C30—C31	113.8 (3)
N3—C7—C6	131.6 (4)	N4—C30—H30A	108.8
N3—C7—C2	106.6 (3)	C31—C30—H30A	108.8
C6—C7—C2	121.8 (4)	N4—C30—H30B	108.8
N3—C8—C9	112.9 (3)	C31—C30—H30B	108.8
N3—C8—H8A	109.0	H30A—C30—H30B	107.7
C9—C8—H8A	109.0	C30—C31—C32	109.7 (4)
N3—C8—H8B	109.0	C30—C31—H31A	109.7
C9—C8—H8B	109.0	C32—C31—H31A	109.7
H8A—C8—H8B	107.8	C30—C31—H31B	109.7
C8—C9—C10	111.5 (3)	C32—C31—H31B	109.7
C8—C9—H9A	109.3	H31A—C31—H31B	108.2
C10—C9—H9A	109.3	C31—C32—C33	113.6 (5)
C8—C9—H9B	109.3	C31—C32—H32A	108.9
C10—C9—H9B	109.3	C33—C32—H32A	108.9
H9A—C9—H9B	108.0	C31—C32—H32B	108.9
C11—C10—C9	112.6 (4)	C33—C32—H32B	108.9
C11—C10—H10A	109.1	H32A—C32—H32B	107.7
C9—C10—H10A	109.1	C34—C33—C32	113.1 (6)
C11—C10—H10B	109.1	C34—C33—H33A	109.0
C9—C10—H10B	109.1	C32—C33—H33A	109.0
H10A—C10—H10B	107.8	C34—C33—H33B	109.0
C10—C11—C12	112.5 (4)	C32—C33—H33B	109.0
C10—C11—H11A	109.1	H33A—C33—H33B	107.8
C12—C11—H11A	109.1	C33—C34—H34A	109.5
C10—C11—H11B	109.1	C33—C34—H34B	109.5
C12—C11—H11B	109.1	H34A—C34—H34B	109.5
H11A—C11—H11B	107.8	C33—C34—H34C	109.5
C11—C12—H12A	109.5	H34A—C34—H34C	109.5
C11—C12—H12B	109.5	H34B—C34—H34C	109.5
H12A—C12—H12B	109.5		
			
C4P—O1P—C1P—C2P	−55.9 (6)	C15—C16—C17—C23	−179.9 (4)
Hg—O1P—C1P—C2P	100.4 (4)	C16—C17—C18—C13	0.3 (5)
C3P—O2P—C2P—C1P	−59.8 (6)	C23—C17—C18—C13	179.3 (3)
O1P—C1P—C2P—O2P	58.5 (6)	C16—C17—C18—Te1	178.0 (3)
C2P—O2P—C3P—C4P	59.4 (7)	C23—C17—C18—Te1	−3.0 (4)
C1P—O1P—C4P—C3P	55.9 (7)	C14—C13—C18—C17	1.5 (5)
Hg—O1P—C4P—C3P	−97.5 (5)	C1—C13—C18—C17	−178.4 (3)
O2P—C3P—C4P—O1P	−58.5 (7)	C14—C13—C18—Te1	−176.3 (3)
C2—N1—C1—N3	1.5 (4)	C1—C13—C18—Te1	3.9 (4)
Te1—N1—C1—N3	−174.9 (2)	C14—C15—C19—C20	−4.6 (5)
C2—N1—C1—C13	−179.5 (3)	C16—C15—C19—C20	176.5 (4)
Te1—N1—C1—C13	4.2 (4)	C14—C15—C19—C21	−124.6 (4)
C7—N3—C1—N1	−1.8 (4)	C16—C15—C19—C21	56.4 (5)
C8—N3—C1—N1	173.3 (4)	C14—C15—C19—C22	115.2 (4)
C7—N3—C1—C13	179.3 (4)	C16—C15—C19—C22	−63.8 (5)
C8—N3—C1—C13	−5.6 (7)	C24—N2—C23—N4	1.5 (4)
C1—N1—C2—C3	−179.8 (4)	Te1—N2—C23—N4	179.4 (2)
Te1—N1—C2—C3	−5.0 (7)	C24—N2—C23—C17	−178.1 (3)
C1—N1—C2—C7	−0.6 (4)	Te1—N2—C23—C17	−0.1 (4)
Te1—N1—C2—C7	174.2 (3)	C29—N4—C23—N2	−0.3 (4)
N1—C2—C3—C4	178.9 (4)	C30—N4—C23—N2	−171.4 (4)
C7—C2—C3—C4	−0.2 (6)	C29—N4—C23—C17	179.2 (4)
C2—C3—C4—C5	1.7 (7)	C30—N4—C23—C17	8.1 (7)
C3—C4—C5—C6	−1.4 (8)	C18—C17—C23—N2	1.9 (5)
C4—C5—C6—C7	−0.4 (7)	C16—C17—C23—N2	−179.1 (4)
C1—N3—C7—C6	−178.2 (4)	C18—C17—C23—N4	−177.6 (4)
C8—N3—C7—C6	6.5 (7)	C16—C17—C23—N4	1.4 (7)
C1—N3—C7—C2	1.3 (4)	C23—N2—C24—C29	−2.1 (4)
C8—N3—C7—C2	−174.0 (3)	Te1—N2—C24—C29	−179.3 (3)
C5—C6—C7—N3	−178.7 (4)	C23—N2—C24—C25	177.3 (4)
C5—C6—C7—C2	1.9 (6)	Te1—N2—C24—C25	0.1 (7)
N1—C2—C7—N3	−0.5 (4)	N2—C24—C25—C26	179.7 (4)
C3—C2—C7—N3	178.9 (4)	C29—C24—C25—C26	−1.0 (6)
N1—C2—C7—C6	179.1 (4)	C24—C25—C26—C27	−0.2 (6)
C3—C2—C7—C6	−1.6 (6)	C25—C26—C27—C28	1.4 (6)
C1—N3—C8—C9	93.8 (5)	C26—C27—C28—C29	−1.2 (6)
C7—N3—C8—C9	−91.8 (5)	C27—C28—C29—C24	0.0 (6)
N3—C8—C9—C10	−173.8 (3)	C27—C28—C29—N4	178.4 (4)
C8—C9—C10—C11	−173.5 (4)	N2—C24—C29—C28	−179.4 (4)
C9—C10—C11—C12	−174.6 (4)	C25—C24—C29—C28	1.2 (6)
N1—C1—C13—C14	174.8 (4)	N2—C24—C29—N4	1.9 (4)
N3—C1—C13—C14	−6.4 (7)	C25—C24—C29—N4	−177.6 (3)
N1—C1—C13—C18	−5.3 (5)	C23—N4—C29—C28	−179.6 (4)
N3—C1—C13—C18	173.5 (4)	C30—N4—C29—C28	−7.9 (6)
C18—C13—C14—C15	−2.5 (6)	C23—N4—C29—C24	−1.0 (4)
C1—C13—C14—C15	177.3 (4)	C30—N4—C29—C24	170.7 (3)
C13—C14—C15—C16	1.8 (6)	C23—N4—C30—C31	−92.9 (5)
C13—C14—C15—C19	−177.2 (4)	C29—N4—C30—C31	97.3 (4)
C14—C15—C16—C17	0.0 (6)	N4—C30—C31—C32	−178.7 (4)
C19—C15—C16—C17	179.0 (4)	C30—C31—C32—C33	171.3 (5)
C15—C16—C17—C18	−1.0 (6)	C31—C32—C33—C34	66.0 (8)

### Hydrogen-bond geometry (Å, º)

**Table:**

*D*—H···*A*	*D*—H	H···*A*	*D*···*A*	*D*—H···*A*
C3—H3*A*···I1	0.95	3.16	4.086 (4)	164
C8—H8*B*···I2^i^	0.99	3.08	3.997 (4)	155
C9—H9*B*···I1^ii^	0.99	3.18	4.095 (4)	155
C30—H30*B*···I1^iii^	0.99	3.28	4.111 (4)	142
C31—H31*B*···I3^iv^	0.99	3.20	4.185 (4)	172

Symmetry codes: (i) *x*, −*y*+3/2, *z*−1/2; (ii) −*x*+1, −*y*+1, −*z*+1; (iii) −*x*+2, −*y*+1, −*z*+1; (iv) −*x*+2, *y*−1/2, −*z*+3/2.
